# P-1547. Real-World Patient Insights Inform an Action-Oriented Healthcare Professional (HCP) Educational Initiative to Address Gaps in Uncomplicated Urinary Tract Infection (uUTI) Management

**DOI:** 10.1093/ofid/ofae631.1714

**Published:** 2025-01-29

**Authors:** Kalpana Gupta, Kara Cossis, Leah Molloy, Laura Simone, Chris Napolitan, Jeffrey D Carter, Jenniffer A Meza Jimenez, Melissa Rodriguez, Chelsie Anderson Chadha, Bonnie Douglas

**Affiliations:** VA Boston HCS and Boston University School of Medicine, Boston, Massachusetts; Chesapeake Urology, Luthervile, Maryland; PRIME Education, Brighton, Michigan; PRIME Education, LLC, Fort Lauderdale, Florida; PRIME Education, Brighton, Michigan; PRIME Education, LLC, Fort Lauderdale, Florida; PRIME Education, Brighton, Michigan; PRIME Education, Brighton, Michigan; PRIME Education, LLC, Fort Lauderdale, Florida; PRIME Education, LLC, Fort Lauderdale, Florida

## Abstract

**Background:**

uUTIs are among the most common outpatient infections and reasons for antibiotic use. This project aimed to understand the real-world burden of UTIs on patients, uncover HCP challenges in uUTI management, and educate HCPs to improve knowledge of guidelines and align clinical practices with patient priorities.

**Table 1.**
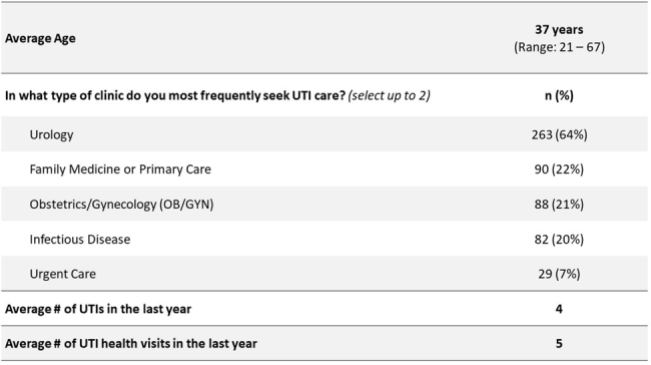
Demographics of Women Who Experience UTIs Who Participated in the Patient Survey (N = 413).

**Methods:**

Patient surveys were distributed nationwide to women who experience UTIs (Table 1). Baseline HCP surveys were administered in 6 clinics that treat UTIs in the southeast US (Table 2). Survey outcomes informed live education sessions with each clinic from 10/2023 – 2/2024, featuring pre-/post-session surveys and team-based action planning.

**Table 2.**
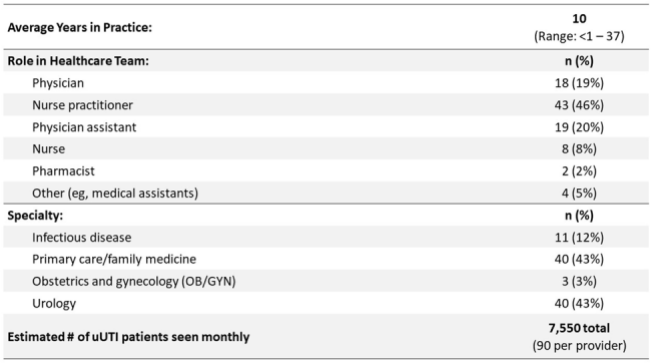
HCP Baseline Survey Participant Demographics (N = 94).

**Results:**

Outcomes from the patient survey revealed the extensive burden of UTIs on quality of life (N = 413, Fig 1). Patients experienced 4 UTIs in the last year on average and reported the most difficult aspect of experiencing UTIs was fear of recurrence (49%), with 79% of patients seen in urology clinics and 66% of patients seen in infectious disease clinics agreeing they were frequently concerned about recurrence versus 58% of patients seen in other clinics (p < .001 and p = .217, respectively). HCPs (N = 94) also reported their top challenge in uUTI care was management of persistent or recurrent infections (54%) and felt they could most improve on antibiotic stewardship (47%) and aligning clinic protocols with the latest guidelines (42%). HCP education sessions led to improvements in knowledge and confidence in uUTI management. After sessions, more HCPs correctly selected 5 days of nitrofurantoin as a first-line uUTI treatment (85% vs 61%, p < .001) and reported high confidence in applying the latest guidelines (72% vs 40%, p < .001) than before. Patient survey data presented to HCPs during sessions showed patients thought their care teams could most improve on counseling about antibiotic side effects (50%) and education about risks of frequent antibiotic use (47%). In response, 93% of HCPs committed to counseling patients about antibiotic use and UTI prevention and clinic action plans included expansion of patient education efforts.

**Figure 1.**
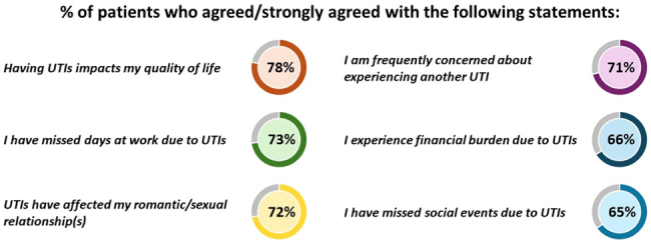
Real-World Patient Insights on UTI Burden.

**Conclusion:**

This initiative offers real-world insights on the burden of UTIs on patients and demonstrates the potential impact of patient-informed education on HCP knowledge and clinical practice in uUTI care.

**Disclosures:**

**Kalpana Gupta, MD MPH**, GlaxoSmithKline: Advisor/Consultant|Iterum Therapeutics: Advisor/Consultant|PhenUtest Diagnostics: Advisor/Consultant|Qiagen: Advisor/Consultant|Up to Date: Royalties|Utility Therapeutics: Advisor/Consultant **Kara Cossis, MPH, PA-C**, Amgen: Honoraria|Astellas: Advisor/Consultant|AstraZeneca: Advisor/Consultant|Bayer: Advisor/Consultant|Janssen: Advisor/Consultant|Myriad: Advisor/Consultant|Tolmar: Advisor/Consultant

